# A Dual‐Mode Photoconductive Colorimetric Wearable UV‐Dosimeter: Strategies for Tunable UV‐Band Sensitivity

**DOI:** 10.1002/smll.74441

**Published:** 2026-07-06

**Authors:** Suman Kundu, Aneeta Jaggernauth, Robert O'Connor, Rajani K. Vijayaraghavan

**Affiliations:** ^1^ School of Electronic Engineering Dublin City University Glasnevin Dublin Ireland; ^2^ School of Physical Sciences Dublin City University Glasnevin Dublin Ireland

**Keywords:** band tunability, colorimetric, metal halides, smart dosimeters, UV detectors

## Abstract

Excessive exposure to ultraviolet radiation poses serious risks to human health, necessitating the development of reliable personal UV monitoring devices. For practical use, such devices must be versatile, UV selective, and capable of providing detailed feedback on cumulative dosage. In this work, thermally evaporated CuBr thin films are employed as UV dosimeters. Owing to charge trapping at CuBr grain boundaries and surface oxide formation, the devices exhibit sustained current increase under prolonged UV exposure. The device response is markedly stronger under UV‐A illumination compared to UV‐B or UV‐C exposure. Wearable UV dosimeters are then constructed by integrating the CuBr films with a custom‐designed battery‐powered circuit, enabling real‐time quantification of ambient solar dosage. Additionally, with increasing solar dose, CuBr films deposited on white ceramic substrates exhibit a distinct color transition from soft amber to dark brown, facilitating dual‐mode UV dosimetry. Wavelength‐tunable responses are also achieved: first, by incorporating a top TiO_2_ layer on CuBr, which filters UV‐B radiation and further enhances the UV‐A selectivity; and second, by introducing mixed compositions of SnBr_2_‐CuBr, which significantly improve UV‐B sensitivity with increasing SnBr_2_ content. This strategy paves the way toward scalable, low‐cost, and wavelength‐selective smart UV dosimeters for applications in personalized and environmental monitoring.

## Graphical abstract

The harmful ultraviolet component of sunlight necessitates reliable and wavelength‐selective personal UV monitoring technologies. Here, thermally evaporated CuBr thin films enable dual‐mode UV dosimetry through persistent photoconductive and colorimetric responses arising from charge trapping and surface oxide formation. Wearable, battery‐powered devices are developed for real‐time solar dose quantification. Further, TiO_2_‐CuBr and SnBr_2_‐CuBr architectures achieve tunable UV‐A and UV‐B selectivity.

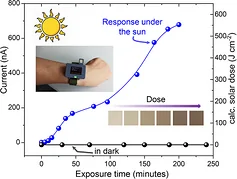

## 1 Introduction

Accurate ultraviolet radiation (UVR) monitoring is critically important across various domains, including personal and occupational health, precision agriculture, environmental monitoring, and industrial processes [1, 2, 3, 4]. While limited UVR exposure is essential for Vitamin D synthesis in the human body, over exposure is the primary cause of skin cancer, the most prevalent cancer globally, and is associated with other health issues such as immunosuppression and cataracts [5, 6]. Notably, approximately 83% of the melanoma cases in individuals aged 30 and above are linked to excessive UV exposure, highlighting the importance of effective control and management of UVR [7]. UVR consists of three spectral bands: UV‐A (315–400 nm), UV‐B (280–315 nm), and UV‐C (100–280 nm), each with distinct biological impacts and applications [8]. While UV‐C and most of UV‐B radiations are absorbed by the stratospheric ozone layer, approximately 94% of solar UVR reaching the earth's surface is UV‐A, with the remaining 6% being UV‐B [9, 10]. UV‐A penetrates deeply into the dermis, whereas UV‐B, though less penetrating, carries higher energy and is more effective in inducing direct DNA damage, primarily through the formation of cyclobutane pyrimidine dimer (CPD), which can lead to mutagenesis and skin cancer [5, 11, 12, 13, 14]. Both UV‐A and UV‐B also generate reactive oxygen species, contributing to oxidative stress and further DNA damage [5, 11, 12, 13, 14]. UV‐C, the most energetic and biologically hazardous UV band, is primarily encountered through artificial sources such as sterilization systems, welding, UV curing, and semiconductor manufacturing processes [15, 16]. In agriculture, UVR plays a dual role, inducing DNA damage and activating the repair mechanism, both of which are wavelength‐dependent [3, 17]. UV‐A and UV‐B have also been shown to differentially influence plant growth, photosynthesis, and plant‐insect interactions, with significant implications for precision agriculture [18, 19, 20, 21]. Environmental factors such as climate conditions, geographical locations, pollution levels, and the presence of reflective surfaces such as water and snow, significantly influence UV intensity [2, 22, 23].

Considering the diverse sources of UVR and its varied biological effects, the development of affordable, compact, and easy‐to‐operate UV dosimeters capable of providing accurate, real‐time monitoring of cumulative UV exposure has become increasingly important [1, 2, 4, 24]. In this context, electronic UV detectors based on wide bandgap semiconductors, whose current varies with UV dose, have been widely studied (Table T1) [25, 26, 27, 28, 29]. Nonetheless, only a small fraction of them possesses cumulative measurement capabilities [29], which avoid the need for continuous sampling and signal processing that are typically required due to the reversible nature of most photoconductors. Colorimetric UV dosimeters that change color upon UV exposure have also emerged as widely adopted alternatives (Table T1) [2, 30, 31, 32, 33, 34]. Yet, their color variations can be subtle and difficult to discern with the naked eye. Therefore, integrating photoconductive and colorimetric functionalities within a single device would be highly advantageous, offering improved accuracy in UV dose detection (Table T1) [35, 36]. However, existing dual‐mode dosimeters either lack cumulative response capability [35] or rely on complex system designs that are unsuitable for smart, wearable applications [36]. Furthermore, the overlapping and often competing effects of different UVR bands present significant challenges for accurate interpretation and risk assessment, emphasizing the need for band‐selective detection; a feature largely overlooked in state‐of‐the‐art dosimeters.

Copper (I) bromide is a wide direct bandgap semiconductor (∼ 3.1 eV), naturally exhibits p‐type conductivity, and is highly transparent (> 80%) across most of the visible spectrum, while strongly absorbing in the UV region [37, 38, 39, 40]. CuBr has attracted significant interest for applications in sensing, solar energy harvesting, and transparent conducting materials [37, 38, 39, 40, 41, 42, 43]. Our group has previously demonstrated a substantial enhancement in photoluminescence and electrical conductivity in CuBr upon UV exposure [37]. In this paper, we present the fabrication of smart, user‐friendly wearable dosimeters, for personal and occupational UV exposure monitoring. The devices employ thermally evaporated CuBr thin films, which exhibit a sustained increase in current and long‐term retention under cumulative UV exposures across different bands, with the current rising by over four orders of magnitude under a very high UV‐A dose of 40 000 mJ cm^−2^. The CuBr thin films can be seamlessly integrated using a custom‐designed circuit to produce smart alerts, prompting users to seek shade before reaching the minimum erythema dose (MED). In addition, these films also demonstrate readily distinguishable color change upon UV exposure, allowing battery‐less operation. Furthermore, we implement novel material compositions and UV filtering layers on CuBr thin films to develop devices with strong spectral selectivity, separately tailored for UV‐A and UV‐B radiations. The present approach enables the development of scalable, low‐cost, versatile, and wavelength‐selective smart UV dosimeters, suitable for applications in personalized and environmental monitoring.

## 2 Results and Discussion

### 2.1 Fabrication of UV Dosimeters and Their Performance Under Different UV Bands

**FIGURE 1:**
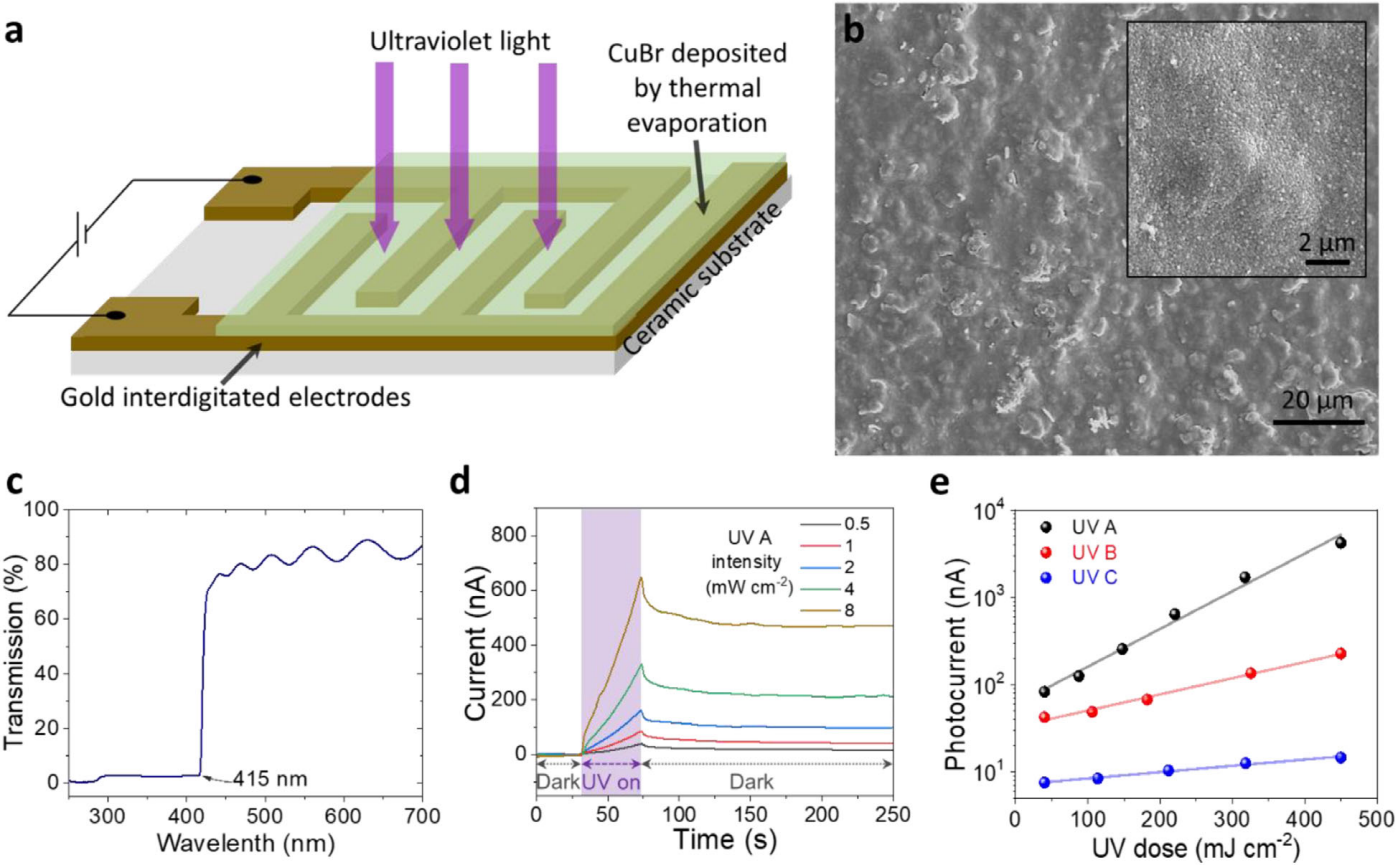
Fabrication of UV dosimeters using thermally evaporated CuBr films. (a) Schematic illustration of the fabricated UV dosimeter. CuBr thin films were deposited onto gold interdigitated electrodes on ceramic substrates via thermal evaporation. The device was exposed to UV radiation, and the current was monitored in real time under a constant 1 V bias. (b) SEM image of the surface of the CuBr thin films. The inset shows a higher magnification of the film surface, showing a densely packed granular morphology. (c) UV–visible transmission spectrum of CuBr thin film (of thickness 970 nm) deposited on quartz substrates. (d) Response of the CuBr‐device under varying intensities (0.5–8 mW cm^−2^) of UV‐A. (e) UV‐induced current profiles of the devices under increasing doses of UV‐A (365 nm), UV‐B (302 nm) and UV‐C (254 nm) radiations.

To fabricate UV dosimeters, thin films of copper (I) bromide were deposited onto ceramic substrates with pre‐patterned gold interdigitated electrodes (gap dimension of 200 µm) by thermal evaporation as illustrated in Figure 1a. Thermal evaporation was selected for its ability to produce uniform, high‐purity films with controllable thickness (see Figure S1). The surface morphology of the thermally evaporated CuBr films was examined using scanning electron microscopy (SEM) as shown in Figure 1b. The images revealed a densely packed granular structure of CuBr, suggesting a polycrystalline growth mode. To assess the optical characteristics of the films, UV‐visible transmission spectroscopy was performed on CuBr films deposited on transparent quartz substrates. The spectrum showed a pronounced decrease in transmittance within the ultraviolet region, particularly below 415 nm (Figure 1c, and Figure S1), which corresponds to the band‐edge of CuBr, associated with its direct bandgap of around 3 eV [37, 38, 39, 40]. This absorption behavior suggests that CuBr films can efficiently absorb photons mainly in the UV region, which can potentially generate charge carriers contributing to photoconductivity.

Our previous study on CuBr‐based devices demonstrated UV‐induced photoconductivity, highlighting their potential for dosimetry applications [37]. However, their performance as UV dosimeters has not yet been comprehensively evaluated, and is an essential step toward assessing their practical viability. In this work, we address this gap by systematically investigating the dosimetry response of CuBr‐based devices under controlled exposure to different UV bands. Devices with a CuBr thickness of ∼ 970 nm were exposed to UV‐A (365 nm), UV‐B (302 nm), and UV‐C (254 nm) radiations for 40 s, each at an intensity of 1 mW cm^−2^ (Figure S2). The device current was monitored in real time under a 1 V bias. Under dark condition, the currents of these devices were around 2.5–3 nA. Upon UV exposures, all devices exhibited increased current response, confirming the UV sensitivity of the CuBr films. The strongest response was observed under UV‐A illumination reaching a peak current of 85 nA, while UV‐B and UV‐C produced weaker responses with peak current values of ∼ 43 and 10 nA, respectively (Figure S2a). This trend could be attributed to the reduced penetration depth of shorter‐wavelength photons (Figure S2b,c), limiting carrier generation within the bulk of the film and increasing susceptibility to surface states and surface recombination centers. We then exposed the device under varying intensities of UV‐A (Figure 1d). As the UV‐A intensity increased from 0.5 to 8 mW cm^−2^ at a fixed exposure time of 40 s, the peak photocurrent rose markedly from 40 to 658 nA. This behavior demonstrates a nearly proportional relationship between light intensity and photocurrent generation. Beyond the immediate response, a notable feature was the persistence of photocurrent even after the UV source was switched off. This long‐retaining behavior, known as persistent photoconductivity (PPC), points to charge trapping mechanisms within the CuBr films. Such behavior is consistent with the high density of grain boundaries observed in SEM images (Figure 1b), which can act as trapping sites for photogenerated carriers [44, 45] as well as the formation of UV‐induced oxide species on the CuBr surface [37]. The presence of PPC makes CuBr particularly attractive for UV dosimetry, as the long‐retained photocurrent can effectively serve as a “memory” of the accumulated radiation dose. We next compared the PPC effect across CuBr films of five different thicknesses‐127, 340, 496, 794, and 970 nm (Figures S3 and S4). All devices exhibited comparable initial dark currents in the range of 2–6 nA (Figure S3). Under both UV‐A and UV‐B illumination, the peak photocurrent increased as the film thickness was raised from 127 to 340 nm. However, with further increase in thickness, the peak current decreased significantly as the carriers are more likely to get recombined or trapped within thicker films before reaching the bottom electrodes. Among all the devices, the 970 nm film exhibited the strongest persistence in its photoresponse (Figure S3), retaining approximately 55% and 62% of its photocurrent under UV‐A and UV‐B illumination, respectively, even 100 s after the light sources were switched off. This behavior can be attributed to the higher probability of photogenerated carrier trapping in thicker films, which enhances charge retention and prolongs the decay of photocurrent. To analyze the decay dynamics of the photocurrent, the transient response was fitted using a double‐exponential model:

(1) $$I_{\mathrm{norm},\mathrm{decay}}=A_{1}e^{-\frac{t}{\tau_{1}}}+A_{2}e^{-\frac{t}{\tau_{2}}}$$

where A_1_ and A_2_ are weighting coefficients and τ_1_ and τ_2_ are decay time constants. From the fitting results (Figure S4), τ_1_ and τ_2_ were extracted, corresponding to the fast and slow charge‐carrier recombination processes, respectively. The fast decay (τ_1_) can be attributed to charges in shallow trap states, whereas the slow process (τ_2_) corresponds to carriers captured in deeper trap states within the CuBr films [46, 47]. As anticipated, the device with a 970 nm CuBr layer displayed the largest τ_2_ value of 404 s with UV‐A and 553 s with UV‐B exposures (Figure S4), confirming the pronounced persistence effect at this thickness, which is beneficial for dosimetry applications. Based on this, the 970 nm device was chosen for detailed characterization in subsequent experiments. Thinner CuBr films (∼ 340 nm) could offer higher UV sensitivity and faster response (Figure S3a–d), but at the expense of reduced retention (Figure S4), whereas thicker films (∼ 970 nm) provide lower power consumption due to smaller current increments (Figure S3a–d) along with enhanced retention. To further assess the dosimetry potential of the devices, we subjected them to prolonged UV irradiation and tracked the evolution of photocurrent over time (Figure 1e, and Figure S5). As expected, the photocurrent increased steadily with longer exposure durations, revealing a clear dose‐dependent response. At a UV dose of 450 mJ cm^−2^, the device reached a photocurrent of 2230 nA under UV‐A illumination, while the responses under UV‐B and UV‐C were noticeably lower, reaching 230 nA and a mere 15 nA, respectively. Prolonged UV irradiation further revealed that the photocurrent scales linearly with the accumulated UV dose (Figure S5). Under UV‐A illumination, the current response continued to increase to 2.6 × 10^4^ nA with doses up to approximately 40 000 mJ cm^−2^, beyond which it reached a plateau (Figure S5). In contrast, under UV‐B exposure with doses upto 3300 mJ cm^−2^, the photocurrent increased steadily to 560 nA, with no indication of saturation within this dose range (Figure S5).

### 2.2 Fabrication of Smart Wearable Dosimeters

**FIGURE 2:**
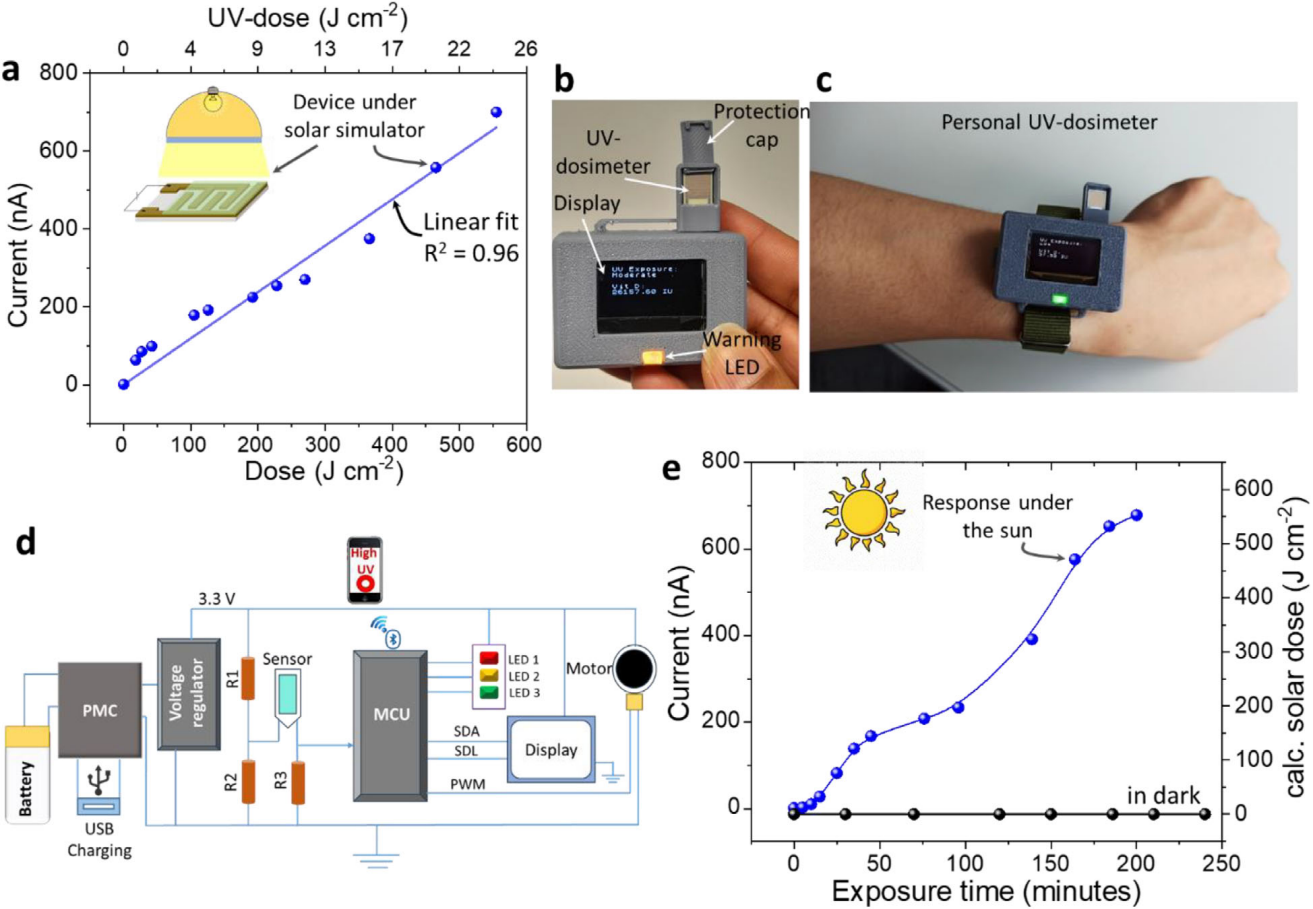
Fabrication of the personal UV dosimeter. (a) Response of the CuBr‐based device under illumination from a solar simulator with an intensity of 1000 W m^−2^, equivalent to standard 1 Sun (AM 1.5G) conditions. The lower y‐axis represents the cumulative dose from the solar simulator across the entire wavelength range, whereas the upper y‐axis indicates the corresponding cumulative UV dose. The response was fitted with a linear function to estimate the rate of current increase. (b, c) Photographs of the smart and wearable personal UV dosimeter. (d) Circuit diagram of the personal UV dosimeter. (e) Photocurrent evolution of the fabricated dosimeter under continuous ambient sunlight. For comparison, the current response of a control device kept in dark conditions is also shown (black line).

To evaluate the real‐world applicability of the CuBr‐based devices for continuous solar monitoring and dosimetry, we examined their photoresponse under simulated solar illumination. The tests were conducted using a calibrated solar simulator with an intensity of 1000 W m^−2^, corresponding to standard 1 Sun conditions (see Figure S6). Upon exposure, the device demonstrated a steady increase in photocurrent as a function of the accumulated light dose (see Figure 2a), confirming a strong correlation between cumulative light dose and current output. The photocurrent rose consistently, reaching approximately 700 nA at a cumulative dose of 550 J cm^−2^. This response is primarily attributed to the UV portion of the solar simulator spectrum (see Figure S6), which corresponds to ∼ 4.3% of the total intensity. To quantitatively assess the dose‐response relationship, a linear fit was applied to the photocurrent data within this dosage range (Figure 2a). The resulting slope provides an estimate of the rate of photocurrent increase with solar exposure. This linearity is a desirable feature for dosimetry applications, as it enables straightforward calibration. To further evaluate the long‐term performance of the device, it was subjected to continuous solar‐simulator irradiation for 7 h, corresponding to a cumulative dose of 2550 J cm^−2^, of which ∼110 J cm^−2^ originated from the UV component (Figure S6). The device continued to exhibit a pronounced increase in photocurrent, ultimately reaching 792 × 10^3^ nA. Notably, the response showed no signs of saturation even at these high cumulative doses. The absence of photocurrent saturation even at elevated exposure levels suggests the device's suitability for long‐term solar dosimetry, where the ability to record cumulative light exposure without signal degradation is essential.

To assess the practical applicability of the prepared devices we made a smart wearable personal UV dosimeter using CuBr films, as shown in Figure 2b–d and Figure S7. This device is capable of measuring real‐time personal UV exposure tailored to specific skin types. According to the Fitzpatrick skin phototype classification, individuals are categorized into skin types I–VI based on their skin's response to UVR. These categories are associated with MED [48], which represent the smallest dose of erythermally‐weighted UVR that produces just‐perceptible skin redness (erythema) [10]. The sensor was calibrated using UV radiation from the solar simulator. The electrical current measured in the absence of UV exposure remained approximately constant and negligible (see Figure 2e) compared to the current measured after exposure. The UV dose was calculated from the known irradiance of the UV lamp and the duration of exposure. Calibration was achieved by correlating the photocurrent range of the sensor (from low to high) with the corresponding MED values. The wearable device is controlled by a microcontroller unit (MCU) and connected as illustrated in Figure 2d. It is powered by a 400 mAh, 3.7 V Li‐ion battery, which is rechargeable via a USB cable. A power management circuit (PMC) handles both charging and power delivery, while a voltage regulator ensures stable voltage during operation. An input voltage of 3.3 V is divided and applied to the sensor, creating a potential difference across it based on its resistance. The resulting photocurrent is measured by the MCU. As UV exposure accumulates, the photocurrent changes accordingly. When the photocurrent reaches the threshold corresponding to the MED for high exposure, the device activates a motor to deliver a warning vibration. The wearable device also features a button that can be pressed at any time to display the current exposure level. This is shown via an LED and display, indicating the words “low,” “medium,” or “high,” along with corresponding colors: green, yellow, or red, based on the cumulative dose. Additionally, the device uses Bluetooth communication to transmit exposure data to a paired smart device (see Figure S8). A dedicated application allows users to input their skin type and view their exposure level through colored rings – green for low, yellow for medium, and red for high, providing a clear visual representation of UV exposure (see Figure S8).

To verify the device's functionality under natural sunlight, experiments were conducted by exposing it to continuous ambient solar conditions. The current followed an increasing trend but with different rates, which can be correlated to the covering of sunlight by clouds as illustrated in Figure 2e. The device‐current reached ∼ 680 nA after 200 min of exposure. Using the earlier established linear fit from Figure 2a, we could estimate the solar dose, and the value was around 540 J cm^−2^ after 200 min of solar exposure. For comparison, a control device maintained under dark conditions (Figure 2e) exhibited only a negligible increase in current (∼ 6 nA) over the same period, confirming that the observed photoresponse is exclusively light‐induced. These results confirm the reliability and consistency of the device's performance under both controlled and real‐world illumination conditions, affirming its potential for cumulative solar and UV dosimetry.

### 2.3 Evaluation of the Colorimetric Variation in CuBr Films Under Solar Exposure

**FIGURE 3:**
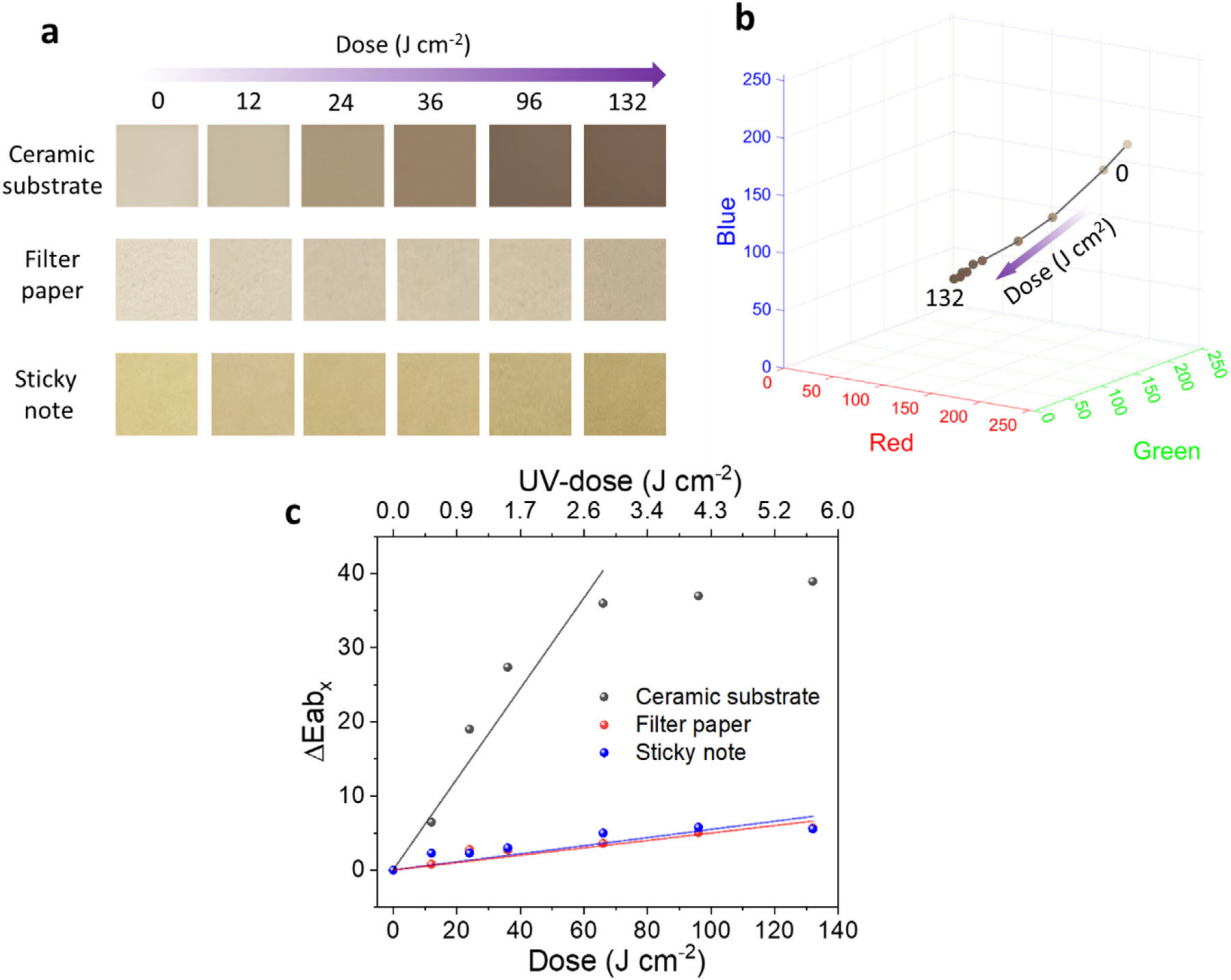
Evaluation of colorimetric dosimeters. (a) The visual images of the CuBr films coated on ceramic substrate, filter paper, and sticky note paper substrate as backgrounds with increasing solar simulator dose. (b) The plot of red, green, and blue coordinates of the CuBr on ceramic substrate with increasing dose. (c) The parameter of color difference (*ΔEab_x_*) of the CuBr films on different substrates with increasing dose from the solar‐simulator. The lower y‐axis represents the cumulative dose from the solar simulator across the entire wavelength range, whereas the upper y‐axis indicates the corresponding cumulative UV dose.

While performing the UV exposure experiments, we observed a distinct change in the visual appearance of the CuBr films, which indicated a possible correlation between light exposure and film coloration. This stimulated our interest in investigating this phenomenon in greater detail, as it could lead to revealing colorimetric UV sensing capability of the CuBr films, further strengthening its UV dosimetry applications. CuBr films were deposited on various substrates and were systematically exposed to simulated solar irradiation using a solar simulator operating at 1000 W m^−2^. The color evolution of the films was monitored against the different surface backgrounds such as white ceramic substrate, white filter paper and sticky note paper of pale‐yellow color, as shown in Figure 3a, Figures S9 and S10. We noticed color changes for all these three substrates, with exposure. The largest change in color was observed for CuBr films deposited on the ceramic substrates where it gradually changed from an initial soft amber to a noticeably darker brown hue, as the cumulative light dose increased from 0 to 132 J cm^−2^ (see Figure 3a and Figure S9). To quantitatively assess the color change, RGB (red, green, blue) color coordinates were extracted from the digital images of the CuBr film‐ceramic samples taken at various light exposure levels using a smartphone‐based application. All three‐color components demonstrated a decreasing trend with increasing light dose (see Figure 3b). The color coordinates on the other background materials, filter paper and sticky note paper exhibited a similar trend but with much smaller variations from their initial values (Figure 3a and Figures S10 and S11). To provide a consolidated metric for quantifying the degree of the color change from the initial values, we derived the parameter of color difference; *ΔEab_x_* using the equation:

(2) $$\Delta Eab_{x}=\sqrt{{\left(\Delta L_{x}^{*}\right)}^{2}+{\left(\Delta a_{x}^{*}\right)}^{2}+{\left(\Delta b_{x}^{*}\right)}^{2}}$$

where, the values of L_x_*, a_x_*, b_x_* were calculated according to the CIE 1976 source = D65 color space parameters (see Tables S1–S3), where L_x_* represents the lightness and a_x_* and b_x_* the color‐opponent dimensions [32, 33]. On the ceramic substrate, the parameter *ΔEab_x_* exhibited a rapid initial increase up to 66 J cm^−2^ dose from the solar‐simulator (corresponding to ∼ 2.84 J cm^−2^ dose in the UV region), which effectively depicted the dose‐dependent colorimetric response of the CuBr film (see Figure 3c). Beyond this point, *ΔEab_x_* gradually stabilized at higher exposure levels. The color variation is likely a result of UV induced chemical modifications within the CuBr layer, potentially involving partial conversion to reddish‐brown copper oxides (Cu_x_O), as observed in our previous studies [37]. Importantly, the color change was readily distinguishable to the naked eye (Figure S9), aided by the high contrast of the bright‐white ceramic substrates. This visual contrast highlights the feasibility of employing CuBr films as standalone, equipment‐free colorimetric indicators for monitoring UV exposure. These findings demonstrate the feasibility of dual‐mode UV dosimetry using CuBr films, which provide both a persistent photoconductive response and a visually perceptible colorimetric change under UV irradiation. This combination offers a distinct advantage over conventional personal UV dosimetry devices, which typically rely on a single detection mechanism. The integration of optical and electrical responses in a single material platform positions CuBr as a promising candidate for next‐generation, low‐cost, and user‐friendly UV dosimeters.

### 2.4 Filtering Incoming UV‐B and Enhancing UV‐A Sensitivity

**FIGURE 4:**
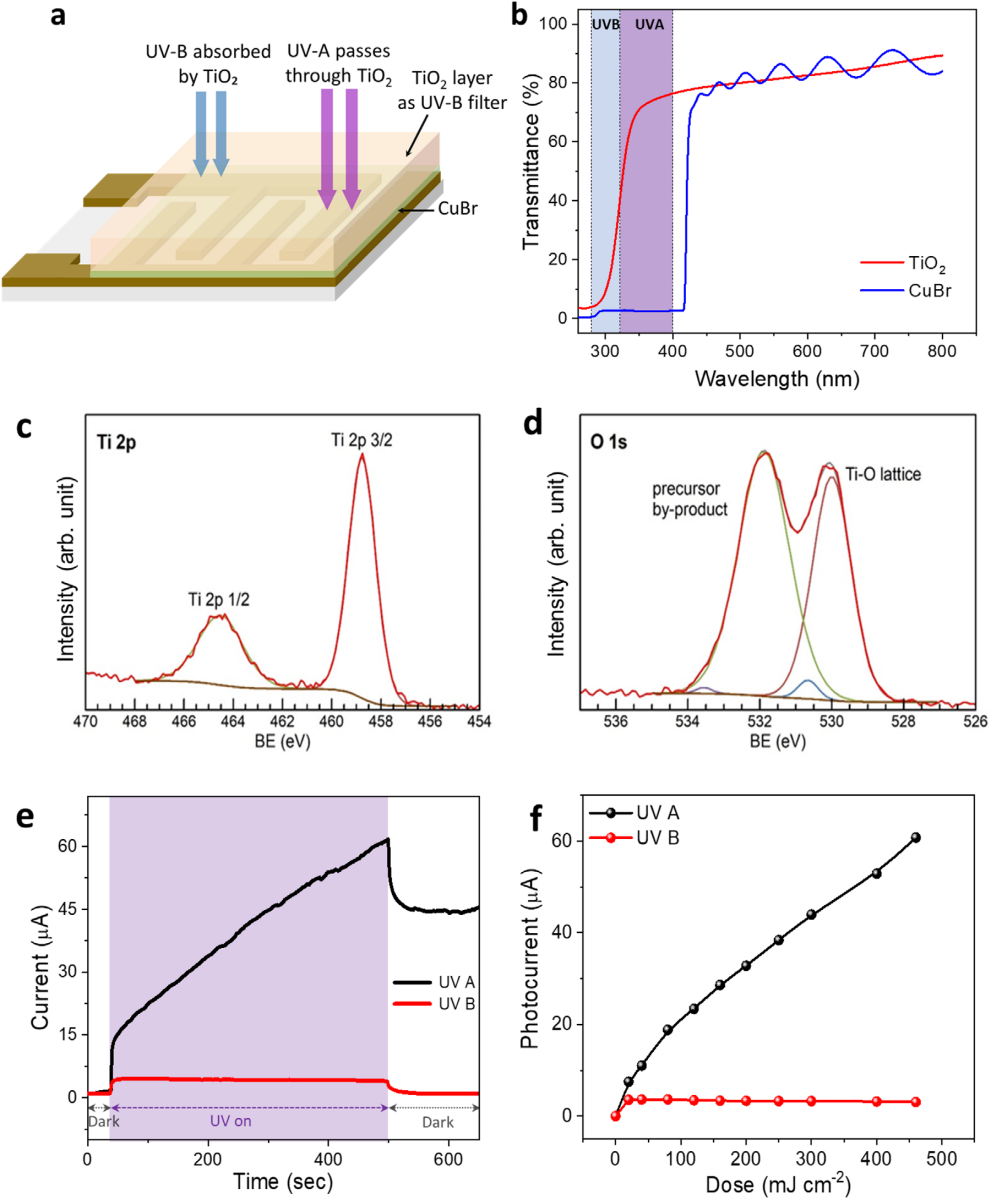
UV‐B filtering using a TiO_2_ layer coated on CuBr. (a) Schematic illustration of the UV‐B filtering with a TiO_2_ layer spin‐coated on CuBr films. (b) The UV‐visible transmission spectra of spin‐coated TiO_2_ film and thermally evaporated CuBr thin film. (c) Ti 2p and (d) O 1s XPS spectra of as‐coated TiO_2_ films. The Ti 2p doublet is fitted with two characteristic peaks for spin‐orbit splitting states 1/2 and 3/2, and the O 1s peak is fitted with two major contributions from the metal oxide lattice at 530.0 eV, and organic species, primarily from Ti precursor reactions, centered at 531.9 eV. (e) Response of the TiO_2_‐coated CuBr device under UV‐A and UV‐B irradiations, each kept at an intensity of 1 mW cm^−2^. (f) The UV induced photocurrents of the device with increasing dose of UV‐A and UV‐B.

While the CuBr films demonstrated higher sensitivity to UV‐A radiation, they also exhibited a significant photocurrent response under UV‐B exposure, which could be detrimental to the applications where only the sensitivity under UV‐A is desired. Thus, to enhance the device's selectivity toward UV‐A and suppress the UV‐B response, a TiO_2_‐based filter layer was introduced, as shown in Figure 4a. TiO_2_ was chosen as the selective UV‐B filtering layer due to its strong and well‐defined absorption in the UV‐B region (< 320 nm), while maintaining high transmittance (> 75%) in the UV‐A (365 nm) and visible ranges (Figure 4b). These features make TiO_2_ an effective material for selectively blocking shorter‐wavelength UVR without interfering with the CuBr film's photoresponse to UV‐A. Additionally, TiO_2_ is chemically stable and compatible with low‐temperature solution‐processing methods, making it ideal for integration with the present CuBr‐based UV dosimeters. TiO_2_ filtering layers were then prepared using a sol–gel method [49]. The precursor solution for the TiO_2_ deposition (see experimental section for details) was stirred for different durations‐4, 6, and 24 h, to study the effect of precursor aging on film properties. These solutions were spin‐coated onto transparent substrates and subsequently annealed at three different temperatures: room temperature (25°C), 100°C, and 350°C to form the TiO_2_ layer. The corresponding UV‐visible transmittance spectra of the resulting films were recorded, as shown in Figure S12. For the 4‐h stirred solution, films with all annealing conditions exhibited a sharp absorption edge below 340 nm, while maintaining high transmittance at longer wavelengths. At longer stirring durations (6 and 24 h), the transmittance spectra began to show noticeable differences. In particular, films prepared from the 24‐h stirred solution exhibited significantly reduced absorption in the UV‐B region, suggesting diminished UV‐B filtering capability. A similar trend was observed when we decreased the spin rate from 1500 to 500 rpm, while keeping the stirring durations fixed at 4 h (see Figure S13). This increment in the UV‐B transmittance was due to micro‐crack network formed in the TiO_2_ layer due to the extended sol aging at prolonged stirring times and higher film thickness at lower spin rates [50], see Figures S13 and S14. Based on these observations, 4‐h stirring and 1000 rpm spin‐rate were selected for preparing the TiO_2_ films as UV‐B filtering layer.

TiO_2_ solution was spin‐coated directly onto the thermally evaporated CuBr films (see Figure 4a) and dried at room temperature to preserve the filtering characteristics without inducing additional thermal effects. To understand the state of coated layers, XPS was performed on the spin‐coated TiO_2_ films to analyze the high‐resolution Ti 2p and O 1s peaks (Figure 4c,d). The Ti 2p doublet peaks were fitted with single peaks positioned at 458.8 and 464.5 eV corresponding to the Ti 2p3/2 and Ti 2p1/2 peaks of tetravalent Ti (Ti^4+^), i.e., TiO_2_. The measured area ratio was 2.1, and peak separation was 5.76 eV [51]. The O 1s peak shows two major contributions. The peak at 530.0 eV is allocated to the Ti─O lattice, and a smaller peak at 530.7 eV suggests the presence of defects in the Ti─O structure or oxide surface terminations [51, 52]. The other major contribution is assigned to unreacted precursor and byproduct species from the reaction. As titanium tetraisopropoxide (Ti(OCH(CH_3_)_2_)_4_) was used as the precursor for the TiO_2_ films, it is likely that organic species remain in the as‐coated film. This broad peak centered at 531.9 eV was thus assigned to organic hydroxide and alkoxy groups [53, 54]. A small contribution from higher binding energy oxides, such as R─C═O, fitted at 533.6 eV, may also result from precursor reactions or surface contamination [54].

The resulting TiO_2_‐coated CuBr devices were then tested under continuous exposure to UV‐A and UV‐B light sources of comparable intensity (1 mW cm^−2^). The initial dark currents of the TiO_2_/CuBr films were around 31 µA; much higher than the pristine CuBr films (2.5–3 nA) due to the introduction of the sol–gel derived TiO_2_ layer. Under UV‐A illumination, the photocurrent increased steadily over time (Figure 4e,f) to 61 µA with increasing dose up to 460 mJ cm^−2^, consistent with the previously observed UV‐A sensitivity of CuBr. Interestingly, under UV‐B exposure, the device showed only an initial sharp increase in the photocurrent to 3.62 µA, which remains stable and gradually decrease to 3 µA with continued exposure (Figure 4e,f). This differs from the pristine CuBr films, where both UV‐A and UV‐B exposures produced similar increasing trend in photocurrent (see Figure 1d,e). The unusual UV‐B response, characterized by an initial increase followed by decay, indicates that interfacial effects play a role in the process (see Figure S15). This behavior confirmed the effectiveness of the TiO_2_ filter in attenuating UV‐B radiation, thereby suppressing the UV‐B induced response in the CuBr layer. These results demonstrate that wavelength‐selective filtering using sol–gel derived TiO_2_ films can be an effective strategy for tailoring the spectral response of CuBr‐based UV dosimeters.

### 2.5 Strategy for Enhancing UV‐B Sensitivity

**FIGURE 5:**
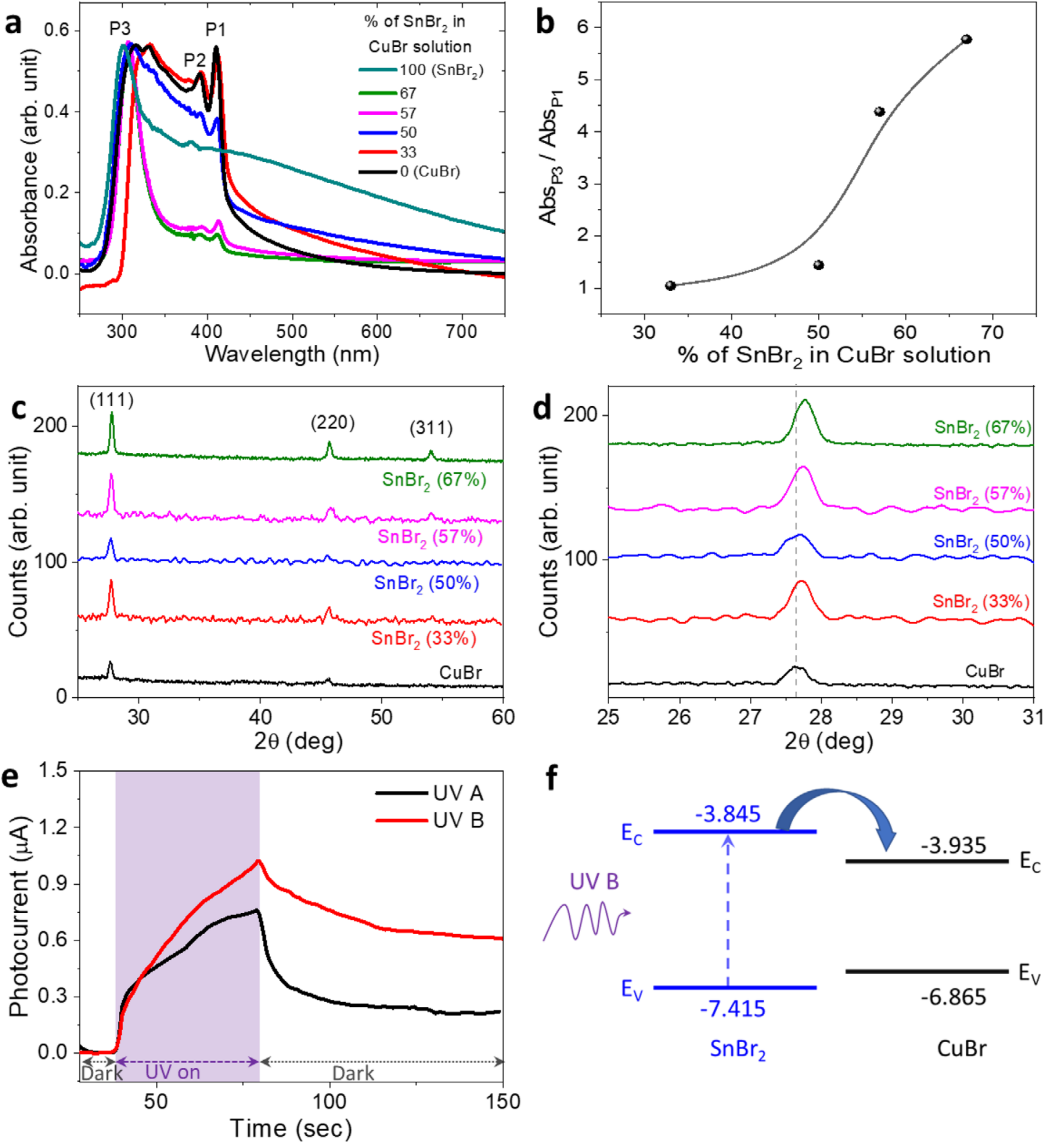
Enhancing UV‐B sensitivity using SnBr_2_:CuBr mixed system. (a) UV‐visible absorption spectra of the films made with various amounts of SnBr_2_ mixed in solutions with CuBr, and annealed at 100°C. Positions of different absorption peaks. (b) The ratios of the absorbance peaks at 315 and 410 nm calculated from (a) for the various mixtures of SnBr_2_ and CuBr. (c, d) The XRD patterns of the as‐fabricated films with mixtures of SnBr_2_ and CuBr. (e) The photocurrent responses of the SnBr_2_:CuBr (1:1) device under UV‐A and UV‐B irradiations, each at an intensity of 1 mW cm^−2^. (f) Schematic showing the energy band diagrams of SnBr_2_ and CuBr with the levels of conduction band minima and valence band maxima indicated. UV‐B radiation is partly absorbed by the SnBr_2_ and generates charge‐carriers which would then transfer to the CuBr, creating an efficient charge transport layer. This phenomenon in‐turn increases the UV‐B sensitivity in the films made with SnBr_2_:CuBr mixtures.

We then aimed to enhance the UV‐B sensitivity of CuBr‐based devices; a feature important for healthcare applications. We hypothesized that integrating CuBr with a material possessing strong UV‐B absorption could improve the overall sensitivity in that region. To implement this strategy, we employed a solution‐processing approach rather than thermal evaporation, allowing homogeneous blending of CuBr with the UV‐B active material, for which we chose SnBr_2_. In this hybrid configuration, CuBr was considered to primarily function as an efficient charge transport layer, facilitating interfacial carrier extraction from the UV‐B absorbing component i.e., from SnBr_2_. To validate the suitability of this solution‐based method, pristine CuBr thin films were first prepared by dissolving CuBr powder in acetonitrile (100 mM) and spin‐coating the solution onto clean quartz substrates. The films were annealed in ambient conditions at temperatures ranging from 100°C to 500°C. UV–visible absorption spectra revealed that CuBr films annealed at 100°C exhibited a distinct absorption edge beginning near 425 nm and two sharp peaks at ∼ 410 nm (P1) and 390 nm (P2) (Figure 5a, and Figure S16), which are associated with the characteristic excitonic Z_1,2_ and Z_3_ peaks of CuBr, respectively [38, 39, 40]. However, as the annealing temperature increased to 300°C, the sharpness of the absorption edge diminished, and at 500°C, the 410 nm peak vanished entirely; suggesting possible thermal degradation of the CuBr films (Figure S16) at higher temperatures than 100°C. To introduce UV‐B sensitivity, SnBr_2_ was selected as the UV‐B active component due to its strong and well‐defined absorption in the UV‐B region, reaching its peak at 300 nm (P3), see Figure 5a and Figure S17. This makes SnBr_2_ an excellent candidate for extending the spectral sensitivity of the CuBr‐device toward UV‐B wavelengths. Additionally, SnBr_2_ is readily soluble in polar solvents like acetonitrile, which enables solution‐based processing compatible with CuBr, allowing for straightforward fabrication of blended films via spin‐coating without the need for complex deposition techniques. Its halide‐based chemistry also aligns well with CuBr, which could support efficient charge transfer between the two materials. SnBr_2_ thin films were prepared via a similar solution‐processing technique using 100 mM solutions in acetonitrile, followed by spin‐coating and annealing over the same temperature range (100°C to 500 °C) as followed for the CuBr films. Across all annealing conditions, SnBr_2_ films consistently exhibited strong UV‐B absorption (see Figure S17). Based on these findings, 100°C‐annealed films were selected for all subsequent experiments due to their appropriate optical characteristics. The bandgaps for the 100°C‐annealed films were estimated using Tauc plot, and the values were 2.93 and 3.57 eV, respectively for CuBr and SnBr_2_ (see Figure S18).

To create a composite system with a tunable spectral response, we prepared mixed SnBr_2_‐CuBr precursor solutions by varying the amount (in %) of SnBr_2_ from 0 (pure CuBr), 33, 50, 57, 67, 100 (pure SnBr_2_). The UV–visible spectra of the mixed films revealed combined features from both compounds, as shown in Figure 5a. Increasing SnBr_2_ content led to a progressive decrease in the 410 nm peak (P1; characteristic of CuBr) and a concurrent increase in the absorbance peak around 315 nm (P3; associated with SnBr_2_), demonstrating successful blending and effective spectral tuning. The ratios of the absorbance peaks P3 and P1 were calculated from Figure 5a for the SnBr_2_‐CuBr mixtures, and the value increased from 1 to 5.8 with increasing the SnBr_2_ amount (see Figure 5b). The X‐ray diffraction (XRD) pattern of these films (shown in Figure 5c), exhibited three distinct peaks at 27.63°, 45.67°, and 53.98° attributed to the (111), (220), and (311) planes of the CuBr structure. Interestingly, we observed the (111) peak gradually shifted from 27.63° to higher 2θ angle up to 27.78° with increasing SnBr_2_ amount (see Figure 5d). The slight shift in the (111) peak is attributed to the lattice strain induced during the formation of the composite film. No additional diffraction peaks corresponding to secondary phases or new compounds were observed in the XRD pattern. Further, the devices fabricated from these mixtures were evaluated similarly under UV‐A and UV‐B illumination (1 mW cm^−2^) for 40 s (Figure S19, Figure 5e). As expected, pristine SnBr_2_ films generated a very small and constant photocurrent (0.2 nA) with UV‐B, which rapidly diminishes when the UV light was turned off, and they did not respond to UV‐A (Figure S19a). Whereas the devices made with SnBr_2_:CuBr mixtures exhibited significant photocurrents under both UV‐A and UV‐B exposures (Figure S19), owing to the inclusion of CuBr. Interestingly, the magnitude of UV‐B‐induced photocurrent was higher or at similar level with UV‐A induced photocurrent with the incorporation of SnBr_2_ in CuBr (Figure S19b–e). With 50%‐SnBr_2_, the UV‐A and UV‐B‐induced photocurrents were around 0.76 and 1.02 µA, respectively (see Figure 5e). This behavior distinctly differs from that of the pristine CuBr films, in which UV‐A induces a higher response than UV‐B (Figure S19f, and Figure 1d,e). Thus, our objective to improve the UV‐B sensitivity of CuBr‐devices was successfully achieved. We then estimated the positions of the valence band maximum and conduction band minimum for SnBr_2_ and CuBr from their electronegativity and bandgap (see section A1 in the Supporting Information) [55, 56]. The calculated band alignment revealed that the energy levels are favorable for the transfer of UV‐B‐generated carriers from SnBr_2_ to CuBr (Figure 5f), resulting in enhanced UV‐B‐induced photocurrent. As the pristine SnBr_2_ film exhibited negligible photocurrent on its own, it primarily functions as a UV‐B absorber, while CuBr serves as an efficient medium for carrier collection and transport. These results further highlight the effectiveness of compositional tuning in mixed‐halide systems to enable wavelength‐selective UV sensitivity. The synergistic interaction between SnBr_2_ as the UV‐B absorber and CuBr as the transport layer provides a promising route for developing low‐cost, broadband UV dosimeters using scalable solution‐processing methods.

## 3 Conclusion

In summary, we developed dual‐mode photoconductive and colorimetric UV dosimeters based on thermally evaporated CuBr thin films, offering smart and versatile approach for personal and occupational UV exposure monitoring. Owing to charge trapping at the grain boundaries of the deposited CuBr and the formation of UV‐induced oxide species on its surface, the devices exhibited persistent photoconductivity, characterized by a continuous increase in UV‐induced current under prolonged exposure and high photocurrent retention even after the UV source was removed. For the as‐deposited CuBr films, the device response under UV‐A illumination was orders of magnitude higher compared to UV‐B and UV‐C. The photocurrent continued to rise, even up to a high UV‐A dose of 40 000 mJ cm^−2^, exceeding the initial dark current by more than four orders of magnitude. Smart wearable UV dosimeters were fabricated by integrating the CuBr films with a custom‐designed, battery‐powered circuit capable of real‐time quantification of ambient solar dosage. In addition, CuBr films deposited on white ceramic substrates exhibited a distinct color change from initial soft amber to dark brown, under increasing solar dose, enabling colorimetric UV dosimetry. Finally, we focused on tuning the wavelength selectivity of the devices, an aspect often overlooked in previous UV dosimeter studies. By spin‐coating a TiO_2_ layer on top of the CuBr, we selectively filtered the UV‐B radiation, thereby enhancing UV‐A specificity of the devices. Furthermore, incorporating a SnBr_2_‐CuBr mixture enhanced the UV‐B sensitivity of the devices, with CuBr functioning as a charge‐transport layer for UV‐B induced carriers generated in SnBr_2_. With 50% SnBr_2_ added to the CuBr solution, the device achieved a photocurrent of approximately 1.02 µA under UV‐B exposure, which is about 1.34 times higher than the response under UV‐A at a similar dose (40 mW cm^−2^). These findings pave the way for the development of versatile, scalable, low‐cost, and wavelength‐selective UV dosimeters suitable for personalized and outdoor environmental monitoring applications.

## 4 Experimental Section

### 4.1 Materials and Thin Film Preparation

CuBr thin films of five different thicknesses were deposited onto quartz substrates and interdigitated electrodes on ceramic substrates (DRP‐IDEAU200‐U50 from Metrohm DropSens) by thermal evaporation of CuBr powder (Sigma–Aldrich 99.999%) similar to the previous reports [37, 38].

TiO_2_ filtering layers were prepared using a sol–gel method [49]. In short, a precursor solution was prepared by dissolving titanium tetraisopropoxide (3.5 mL) in isopropanol (25 mL), followed by the addition of a small amount of concentrated HCl (0.1 mL) and distilled water (0.2 mL) to promote hydrolysis and condensation reactions. All materials were purchased from Sigma–Aldrich. These solutions were spin‐coated onto quartz substrates and on CuBr‐coated interdigitated electrodes, and subsequently annealed to form the TiO_2_ layer.

SnBr_2_‐CuBr composite films were prepared using the precursors created by mixing different amounts of CuBr (100 mM) and SnBr_2_ (100 mM) solutions dissolved in acetonitrile and thereafter spin‐coating the mixed solutions onto clean quartz substrates and interdigitated electrodes. All materials were purchased from Sigma–Aldrich.

### 4.2 Thin Film Characterization

X‐ray diffraction analysis was carried out (Bruker D8, θ–2θ configuration) to determine the crystal structure of the deposited thin films. The UV‐visible absorption/transmission properties of the films were studied by a Perkin Elmer Lambda 40 UV/Vis instrument. X‐ray photoelectron spectroscopy (XPS) analysis was carried out on a Scienta Omicron XPS (monochromatic Al Kα 1486.7 eV x‐ray source, base pressure 5 × 10^−9^ mbar) with a 128 channel Argus CU detector (54.7° source analyzer angle). All peaks were referenced to the C 1s peak at 284 eV.

### 4.3 I‐V Characterization

Current from the CuBr‐coated interdigitated electrodes were monitored with 1V bias (using a Keithley 2400 Source Meter and recorded using SweepMe!) under UV light sources of different bands i.e., UV‐A (365 nm), UV‐B (302 nm) (EL series, UVP) and UV‐C (255 nm) (Edmund Optics) and also under a solar simulator (Newport) kept at 1 sun intensity (1000 W m^−2^). UV intensities were calibrated using PM400 optical power meter with a S130VC photodiode (Thorlabs).

## Conflicts of Interest

The authors declare no conflicts of interest.

## Supporting information




**Supporting File**: smll74441‐sup‐0001‐SuppMat.docx.

## Data Availability

The data that support the findings of this study are available from the corresponding author upon reasonable request.
