# Supplementary material for: A Dual‐Mode Photoconductive Colorimetric Wearable UV‐Dosimeter: Strategies for Tunable UV‐Band Sensitivity
Source: Small. 2026 Jul 6;22(48):e74441. doi: 10.1002/smll.74441 (PMC13509126; doi:10.1002/smll.74441)
Supplement: Supplementary file 1 [file SMLL-22-e74441-s001.docx]

Supporting Information

**A dual-mode photoconductive colorimetric wearable UV-dosimeter: strategies for tunable UV-band sensitivity**

Suman Kundu^1^, Aneeta Jaggernauth^1^, Rob O'Connor^2^ and Rajani K Vijayaraghavan^1^*

^1^School of Electronic Engineering, Dublin City University, Glasnevin, Dublin 9, Ireland

^2^School of Physics, Dublin City University, Glasnevin, Dublin 9, Ireland

[*Rajani.vijayaraghavan@dcu.ie](mailto:*Rajani.vijayaraghavan@dcu.ie)

**Table T1.** Comparison of the performance of state-of-the-art photoconductive and colorimetric UV dosimeters

| **Ref. No.** | **UV active material** | **Mode of UV detection** | **Wavelength range for detection** | **Dynamic range** | **Wavelength selectivity** | **ΔE**  **(Parameter of Colour difference)** |
| --- | --- | --- | --- | --- | --- | --- |
| 1. | PVP-functionalized CNT | Photoconductive | UV-A to  UV-C | 90 mJ cm^-2^ under UV-C | Higher responsive to UV-C | - |
| 2. | DTEC/LDPE | Colorimetric | UV-A to  UV-C | 282 mJ cm^-2^ under UV-B | Higher responsive to UV-C | - |
| 3. | PMMA doped with diarylethene (DAE) dyes | Colorimetric | UV-A to  UV-B | 0.15 J cm^-2^ under UV | - | ~ 0 - 50 |
| 4. | PPDPS-TF and Congo red mixed into a PDMS matrix | Colorimetric | UV-A, and UV-B | 0.8 J cm^-2^ under UV-A | Selective to UV-A and UV-B | 0 - 69.73 |
| 5. | BMS:Fe/Eu | Colorimetric | UV-A (405 nm) | 32.9 mJ cm^-2^ under UV-A (405 nm) | Selective to UV | - |
| 6. | WO_3_/PAAM | Photoconductive and colorimetric | UV | 40 mWcm^-2^ under UV | Selective to UV | 0 - 48.2 |
| 7. | PDMAA-Mo(VI) | Photoconductive and colorimetric | UV | 100 J cm^-2^ under UV | Selective to UV | 64.38 - 2.22 |
| **This work** | CuBr thinfilms | Photoconductive and colorimetric | UV-A to  UV-C | 40 J cm^-2^ under UV-A & 3.3 J cm^-2^ under UV-B | CuBr is higher responsive to UV-A. Responses can be tailored for specifically UV-A and UV-B radiations | 0 - 38.94 |

**Figure S1.** (a) Thickness of thermally evaporated CuBr thin films with different amounts of CuBr used for the deposition. (b) UV–visible transmission spectra of CuBr thin films of different thicknesses 127-970 nm.

**Figure S2**. (a) Response of the CuBr-device (of thickness 970 nm) under UV-A (365 nm), UV-B (302 nm) and UV-C (254 nm) irradiations for 40 s, each at an intensity of 1 mW cm^-2^. (b) Absorbance (A) of the CuBr film of thickness (t): 970 nm. (c) Absorption coefficient (α = 2.303*A/t) in nm^-1^ and penetration depth δ (=1/α) in nm derived from b. The average value of δ in UV-A range (400 -315 nm) is ~ 266 nm, in UV-B range (315 - 280 nm) is ~ 251 nm and in UV-C range (280 – 250 nm) is ~ 186 nm.

**Figure S3.** Response of the CuBr-devices with different thicknesses under (a) UV A and (b) under UV B irradiation for 40 seconds, at a fixed intensity of 1 mW cm^-2^. The peak photocurrents of the devices at 40 s exposure for (c) UV-A, and (d) UV-B. The normalised responses of the devices under (e) UV-A and (f) UV-B.

**Figure S4.** (a) Normalised decay response of the photocurrent after 40 s of UV-A exposure (1 mW cm^-2^) in devices with varying thicknesses of CuBr. The curves are fitted with a double exponential function (dashed lines); I_norm,decay_ = A_1_ exp(-t/τ_1_) + A_2_ exp(-t/τ_2_). (b) The derived values of τ_1_ and τ_2_ from a. (c) The normalised decay response of the photocurrent after 40 s of UV-B exposure (1 mW cm^-2^) in devices with varying thicknesses of CuBr. (d) The derived values of τ_1_ and τ_2_ from c.

**Figure S5.** Response of the CuBr-devices (thickness ~ 970 nm) under continuous exposure of (a) UV-A and (b) UV-B.

**Figure S6.** (a) Image of the UV-sensor kept under a solar simulator. Zoomed-in image of the sensor is shown inside. (b) Response of the sensor exposed under the solar-simulator of intensity 1000 W m^-2^. For comparison the current of the device kept in dark-condition is also plotted. (c) Spectral irradiance of the solar simulator in the 250-1100 nm wavelength range.

**Figure S7.** (a) Image of the CuBr-coated interdigitated electrode as the sensor-unit. (b) Image of the sensor-unit under the protective enclosure. (c) Image of the sensor-unit plugged-inside the measurement-unit.

**Figure S8.** Screenshots of the smartphone application indicating different exposure levels for skin type 1 measured with the personal dosimeter.

**Figure S9.** (a-f) Images of the CuBr-coated ceramic substrate under increasing solar simulator exposure dose (0-132 J cm^-2^) as indicated above each figure.

**Figure S10.** (a-f) Images of the CuBr-coated filter paper and sticky note under increasing solar simulator exposure dose (0-132 J cm^-2^) as indicated above each figure. Some regions of the substrates are intentionally kept uncoated with CuBr to serve as references.

**Figure S11.** (a) The plot of red, green and blue coordinates of the CuBr film on (a) filter paper, and (b) sticky note under increasing solar simulator exposure dose (0-132 J cm^-2^).

**Table S1.** Calculated values of *L_x_*, a_x_*, b_x_** and *ΔEab_x_* for CuBr films coated on ceramic substrate.

| Dose  (J cm^-2^) | L_x_* | a_x_* | b_x_* | ΔEab_x_ |
| --- | --- | --- | --- | --- |
| 0 | 81.71 | 0.49 | 11.36 | 0 |
| 12 | 76.23 | -0.03 | 14.71 | 6.45 |
| 24 | 63.97 | 1.5 | 18.08 | 19 |
| 36 | 55.21 | 5.62 | 15.92 | 27.37 |
| 66 | 46.54 | 8 | 12.36 | 35.98 |
| 96 | 45.06 | 5.19 | 12.57 | 36.97 |
| 132 | 43.22 | 6 | 13.58 | 38.94 |

**Table S2.** Calculated values of *L_x_*, a_x_*, b_x_** and *ΔEab_x_* for CuBr films coated on filter paper.

| Dose  (J cm^-2^) | L_x_* | a_x_* | b_x_* | ΔEab_x_ |
| --- | --- | --- | --- | --- |
| 0 | 82.58 | -0.56 | 12.07 | 0 |
| 12 | 82.12 | -1.08 | 12.46 | 0.8 |
| 24 | 82.28 | -0.6 | 14.84 | 2.78 |
| 36 | 82.19 | -0.96 | 14.71 | 2.7 |
| 66 | 80.56 | -0.22 | 15.04 | 3.61 |
| 96 | 78.58 | 0.7 | 14.88 | 5.05 |
| 132 | 77.85 | 0.71 | 14.91 | 5.66 |

**Table S3.** Calculated values of *L_x_*, a_x_*, b_x_** and *ΔEab_x_* for CuBr films coated on sticky note.

| Dose  (J cm^-2^) | L_x_* | a_x_* | b_x_* | ΔEab_x_ |
| --- | --- | --- | --- | --- |
| 0 | 78.38 | -3.04 | 26.86 | 0 |
| 12 | 76.79 | -1.84 | 25.73 | 2.29 |
| 24 | 76.79 | -1.84 | 25.73 | 2.29 |
| 36 | 76.69 | -0.66 | 26.14 | 3 |
| 66 | 74.4 | -1.35 | 29.35 | 4.99 |
| 96 | 73.42 | -0.68 | 28.55 | 5.75 |
| 132 | 73.58 | -1.73 | 29.28 | 5.54 |

**Figure S12.** UV-visible transmittance spectra of TiO_2_ films prepared with stirring time of (a) 4, (b) 6 and (c) 24 hours for titanium tetraisopropoxide in isopropanol. The films were prepared at different annealing temperatures (25 – 350 °C) as indicated inside.

**Figure S13.** (a) UV-visible transmittance spectra of TiO_2_ (with 4 hours stirring time) spin-coated films prepared with different spin rates (500 - 1500 rpm). (b) The thickness of the prepared films.

**Figure S14.** SEM images of the TiO_2_ films coated on CuBr with spin rates (a) 1000 rpm and (b) 500 rpm.

**Figure S15.** XPS analysis of (a) Ti 2p and (b) O 1s spectra from the TiO_2_/CuBr sample after UV-B exposure.

As shown in Figure S15a, the Ti 2p_3/2_ peak comprised of two components separated by ~ 0.6 eV, the larger of which was fitted at the lower BE of 458.8 eV, and the smaller contribution at 459.4 eV are both associated with the Ti^4+^ oxidation state. The small binding energy shift suggests that the Ti atoms exist in two slightly different local chemical environments rather than different oxidation states (as Ti^4+^/Ti^3+^ separation typically exceeds 1.0 eV). Furthermore, the O 1s spectrum (Figure S15b) reveals that the lattice oxygen accounts for only ~55% of the total signal. A substantial contribution (30%) is observed at 531.6 eV corresponding to the hydroxyl groups, suggesting considerable surface hydroxylation under UV-B exposure. These results provide strong evidence that UV-B irradiation induces a modification in the chemical environment of the TiO_2_ layer. The observed variations in local coordination and the prevalence of hydroxyl-related states likely introduce shallow trap states within the near-surface and interfacial regions. Such states can modulate carrier trapping and recombination dynamics, providing a plausible physical explanation for the transient UV-B response (initial rise followed by decay, as observed in Figure 4e,f.

**Figure S16.** UV-visible absorption spectra of CuBr spin-coated films annealed at different temperatures 100-500 °C as indicated inside.

**Figure S17.** The UV-visible absorption spectra of SnBr_2_ spin-coated films annealed at different temperatures 100-500 °C as indicated inside.

**Figure S18**. Tauc plot of 100°C annealed (a) CuBr and (b) SnBr_2_ films. The bandgap (E_g_) values are indicated inside the figures.

**Figure S19.** (a-f) Responses of the devices made with different amounts of SnBr_2_ in CuBr as indicated above each figure, under UV A and UV B exposures with intensity 1 mW cm^-2^.

**Section A1. Theoretical calculation of band positions of CuBr and SnBr_2_ at vacuum scale:**

At vacuum scale, the conduction band minimum (E_C_) and valence band maximum (E_V_) are positioned relative to the vacuum energy level (0 eV). The values of electron affinity (A), electronegativity (χ), and ionization energy (I) can be used to calculate the positions of E_C_ and E_V_.

For a semiconductor the bulk electronegativity (χ) corresponds to the halfway between the bottom of the conduction band and the top of the valance band. ^[8,9]^

χ = A + 0.5 Eg …………… (1)

Thus, the relationship between the band energies and electronegativity can be expressed as below:

E_C_ = - A = - χ + 0.5 Eg	…………… (2)

E_V_ = - I = - χ - 0.5 Eg	…………… (3)

Electronegativity for an element can be calculated as below:

χ = ½ (A + I) …………… (4)

Electronegativity for a compound (M_a_X_b_) can be considered as:

χ = (χ_M_^a^ × χ_N_^b^)^1 / (a + b)^	…………… (5)

The electron affinity of Sn, Br and Cu are determined below using equation 4:

Sn → A = 1.1121, I = 7.3 → χ_Sn_ = 4.21

Br → A = 2.51, I = 10.51 → χ_Br_ = 6.51

Cu → A = 1.23, I = 7.92 → χ_Cu_ = 4.48

Thereafter, the electron affinity of SnBr_2_ and CuBr can be calculated using the equation 5:

- χ of SnBr₂:

χ = (χ_Sn_ × χ_Br_ × χ_Br_)^(1/3)^

= (4.21 × 6.51 × 6.51)^(1/3)^

= 5.63

- χ of CuBr:

χ = (χ_Cu_ × χ_Br_)^(1/2)^

= (4.48 × 6.51)^(1/2)^

= 5.4

Now, the positions of E_C_ and E_V_ of SnBr_2_ and CuBr can be calculated using equations 2 and 3.

- For SnBr_2_:

E_g_ of SnBr_2_ = 3.57 eV (see Figure S18)

E_C_ of SnBr_2_:

E_C_ = -χ + 0.5E_g_

= -5.63 + 0.5 × 3.57 = -3.845 eV

E_V_ of SnBr_2_:

E_V_ = E_C_ - E_g_

= -3.845 - 3.57 = -7.415 eV

- For CuBr:

E_g_ of CuBr = 2.93 eV (see Figure S18)

E_C_ of CuBr:

E_C_ = -χ + 0.5E_g_

= -5.4 + 0.5 × 2.93 = -3.935 eV

E_V_ of CuBr:

E_V_ = E_C_ - E_g_

= -3.935 – 2.93 = -6.865 eV

**References**

1. Yi, Ying, Hui Liao, Xueyan Chang, Xiaochi Liu, Yahua Yuan, and Jian Sun. "An In‐Memory Personal Dosimeter Based on UV Radiation‐Programmed Nonvolatile Memory." *Advanced Functional Materials* (2025): e10064. <https://doi.org/10.1002/adfm.202510064>
2. Wang, Junxin, Ananthakrishnan Soundaram Jeevarathinam, Anamik Jhunjhunwala, Haowen Ren, Jeanne Lemaster, Yanqi Luo, David P. Fenning, Eric E. Fullerton, and Jesse V. Jokerst. "A wearable colorimetric dosimeter to monitor sunlight exposure." *Advanced Materials Technologies* 3, no. 6 (2018): 1800037. <https://doi.org/10.1002/admt.201800037>
3. Butterfield, Jesse L., Jennifer R. Quigley, and Carson J. Bruns. "Smart tattoos of intradermally implanted photochromic nanosensors for personal UV sensing and dosimetry." *Advanced Functional Materials* 34, no. 40 (2024): 2405831. <https://doi.org/10.1002/adfm.202405831>
4. Araki, Hitoshi, Jeonghyun Kim, Shaoning Zhang, Anthony Banks, Kaitlyn E. Crawford, Xing Sheng, Philipp Gutruf, Yunzhou Shi, Rafal M. Pielak, and John A. Rogers. "Materials and device designs for an epidermal UV colorimetric dosimeter with near field communication capabilities." *Advanced Functional Materials* 27, no. 2 (2017): 1604465. <https://doi.org/10.1002/adfm.201604465>
5. Shao, Jie, Shuo Feng, Meng Liu, Yu Guo, Chunxiao Lu, Shanjun Ke, Haiqin Sun et al. "Promising Coloration Composite Strategy to Multicolor Photochromism for On‐Demand Information Displays." *Advanced Functional Materials* (2025): 2425059. <https://doi.org/10.1002/adfm.202425059>
6. Xia, Hongbo, Zikang Wu, Qiyue Tang, and Zijian Yang. "Dual-Response UV Radiation Detector Based on Color Switching and Photoresistance Response for UV Radiation Monitoring." *ACS Applied Materials & Interfaces* 17, no. 3 (2025): 5382-5390. <https://doi.org/10.1021/acsami.4c19617>
7. Wen, Guo-Yu, Xing-Long Zhou, Xiao-Yu Tian, Ting-Yuan Hu, Rui Xie, Xiao-Jie Ju, Zhuang Liu, Da-Wei Pan, Wei Wang, and Liang-Yin Chu. "Real-time quantitative detection of ultraviolet radiation dose based on photochromic hydrogel and photo-resistance." *Chemistry of Materials* 34, no. 17 (2022): 7947-7958. <https://doi.org/10.1021/acs.chemmater.2c01761>
8. Xu, Yong, and Martin AA Schoonen. "The absolute energy positions of conduction and valence bands of selected semiconducting minerals." *American mineralogist* 85, no. 3-4 (2000): 543-556. <https://doi.org/10.2138/am-2000-0416>
9. Butler, M. A., and D. S. Ginley. "Prediction of flatband potentials at semiconductor‐electrolyte interfaces from atomic electronegativities." *Journal of the Electrochemical* Society 125, no. 2 (1978): 228. DOI 10.1149/1.2131419
